# Safety and benefit of using a virtual bolus during treatment planning for breast cancer treated with arc therapy

**DOI:** 10.1002/acm2.12398

**Published:** 2018-06-30

**Authors:** Marguerite Tyran, Agnes Tallet, Michel Resbeut, Marjorie Ferre, Veronique Favrel, Pierre Fau, Laurence Moureau‐Zabotto, Julien Darreon, Laurence Gonzague, Ahcene Benkemouche, Leonel Varela‐Cagetti, Naji Salem, Bertrand Farnault, Marie‐Aimee Acquaviva, Hugues Mailleux

**Affiliations:** ^1^ Department of Radiation‐Oncology Institut Paoli‐Calmettes Marseille France

**Keywords:** breast, radiotherapy, skin flash, virtual bolus, VMAT

## Abstract

**Purpose:**

This study evaluates the benefit of a virtual bolus method for volumetric modulated arc therapy (VMAT) plan optimization to compensate breast modifications that may occur during breast treatment.

**Methods:**

Ten files were replanned with VMAT giving 50 Gy to the breast and 47 Gy to the nodes within 25 fractions. The planning process used a virtual bolus for the first optimization, then the monitors units were reoptimized without bolus, after fixing the segments shapes. Structures and treatment planning were exported on a second scanner (CT) performed during treatment as a consequence to modifications in patient's anatomy. The comparative end‐point was clinical target volume's coverage. The first analysis compared the VMAT plans made using the virtual bolus method (VB‐VMAT) to the plans without using it (NoVB‐VMAT) on the first simulation CT. Then, the same analysis was performed on the second CT. Finally, the level of degradation of target volume coverage between the two CT using VB‐VMAT was compared to results using a standard technique of forward‐planned multisegment technique (Tan‐IMRT).

**Results:**

Using a virtual bolus for VMAT does not degrade dosimetric results on the first CT. No significant result in favor of the NoVB‐VMAT plans was noted. The VB‐VMAT method led to significant better dose distribution on a second CT with modified anatomies compared to NoVB‐VMAT. The clinical target volume's coverage by 95% (V95%) of the prescribed dose was 98.9% [96.1–99.6] on the second CT for VB‐VMAT compared to 92.6% [85.2–97.7] for NoVB‐VMAT (*P = *0.0002). The degradation of the target volume coverage for VB‐VMAT is not worse than for Tan‐IMRT: the median differential of V95% between the two CT was 0.9% for VMAT and 0.7% for Tan‐IMRT (*P* = 1).

**Conclusion:**

This study confirms the safety and benefit of using a virtual bolus during the VMAT planning process to compensate potential breast shape modifications.

## 1 INTRODUCTION

Adjuvant radiotherapy (RT) for breast cancer is a standard treatment used to improve local tumor control and overall survival.^1^, ^2^, ^3^, ^4^ Volumetric modulated arc therapy (VMAT) has been evaluated for breast treatment in several publications as attested in a recent review.^5^ For now, only six publications report a clinical experience dominated by simultaneous integrated boost studies^6^, ^7^, ^8^, ^9^ or accelerated partial breast irradiation^10^ and only one in the setting of nodal involvement.^11^ The location of the mammary gland leads to clinical target volumes (CTV) adjoined to the skin. This particularity of breast's target volume would generate a planning target volume (PTV) located partially outside the external body contour if isotropic margins were applied. In inverse planning optimization, this prevents from taking isotropic PTV margins. It may lead to target volume's lack of coverage in case of inter‐ and/or intrafraction movements. This issue can be taken into account using a skin flash method for fixed fields. However, for arc therapy techniques, other solutions should be found.

A method using virtual bolus to force the leaves to be positioned away from the external part of the breast has been described.^12^ However, it was tested in a theoretical way as automatic expansions were used to mimic inter‐ and/or intrafraction modifications. In this dosimetric study, we use a nearby similar virtual bolus method to check its reliability in true life: We use real setup errors and breast's shape modifications of 10 real patients treated for left breasts and lymph nodes including the internal mammary chain (IMC). These patients had been reimaged with a second scanner (CT) because of observed interfractional modifications.

In order to validate the safety of using of the virtual bolus technique for the inversed optimization process, this paper first evaluates the consequences of using of a virtual bolus on the initial planning CT. Then, the treatment planning reproducibility is investigated by comparing the plans made with the virtual bolus method (VB‐VMAT) to the plans without using it (NoVB‐VMAT) on the second CT. The consequences of these modifications on the coverage of target volumes and dose to the organs at risk are evaluated. Finally, the level of degradation of target volume coverage between the two CT using VB‐VMAT is compared to results using our institutional standard technique of forward‐planned multisegment technique (Tan‐IMRT).

## 2 MATERIALS AND METHODS

### 2.A Patient selection, contouring and prescription

Ten planning studies were performed for patients consecutively treated for breast cancer in our department. Inclusion criteria were left‐sided breast cancer, with a target volume including breast/chest wall, supraclavicular, axillary II and III nodes, and IMC in the first three interspaces, planned on the same version of Pinnacle^®^. Eight patients were treated with breast conservative surgery and two with mastectomy. The same datasets and contours were used to do the standard plans (on Pinnacle^®^ v9.10) and VMAT plans (on RayStation^®^ v5.0). Dose calculations were made using a collapsed cone convolution algorithm on both treatment planning systems (TPS) with a grid size of 3.0 mm. Both the plans were optimized for an Elekta Synergy LINAC equipped with an Agility 160 multileaves collimator (MLC). All patients had a second CT during treatment because of unsatisfying portal images or clinical edema.

**Figure 1:**
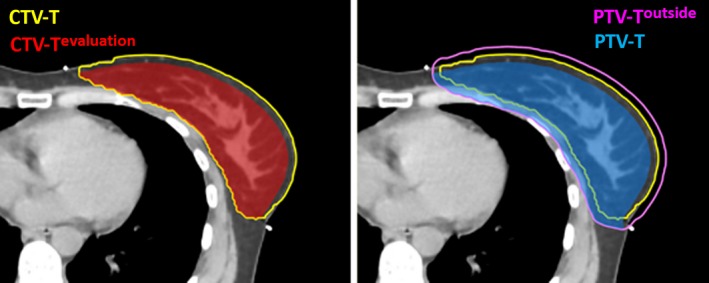
Target volumes creation: The clinical target volume including the breast/chest wall (CTV‐T) is displayed in yellow. The clinical target volume limited 5 mm inside the external contour (CTV‐T^evaluation^) is in red. PTV‐T outside in pink stands for the planning target volume generated by adding a 5 mm margin around the CTV‐T. PTV‐T in blue is constructed by limiting the volume of PTV‐T outside 5 mm inside the skin.

The CTV included the breast/chest wall (CTV‐T), supraclavicular, axillary level II and III nodes, and the IMC in the first three intercostal spaces (CTV‐N). By adding a 5 mm margin around the CTVs, we generated PTVs located partially outside the external body contour. We called those volumes PTV‐T^outside^ and PTV‐N^outside^. Then, by limiting those volumes 5 mm inside the external contour, we created PTV‐T and PTV‐N. Furthermore, to evaluate the degradation of the target coverage on the second CT, a CTV‐T^evaluation^, limited 5 mm inside the external contour, is also constructed (Fig.* * 1).

Prescribed doses were 50 Gy in 25 fractions for the breast/chest wall and 47 Gy in 25 fractions for the regional nodes (corresponding to 46 Gy in 23 f with α/β = 4). The irradiation of the tumor bed is not considered in this work. The organ at risk (OAR) (lungs, heart, left coronary artery (LCA), right breast, humeral head, thyroid, and esophagus) were also contoured according to the RTOG recommendations (http://rtog.org/). A structure called “skin,” was constructed as the 5 mm fringe under the external contour inside the PTV.

Treatment planning and contouring from the first scanner (CT1) were exported on the second scanner (CT2) after registration. These patients had been reimaged during their treatment because of observed interfractional modifications. As described in a previous study,^13^ we used a rigid registration between CT1 and CT2 by focusing on a cubic region including the treated breast. Contours were manually adjusted to the new anatomy and the plan from CT1 was recalculated on CT2 without further optimization.

### 2.B Volumetric Modulated Arc Therapy

For the VMAT plans, we used two arcs starting from 300° to 170° clockwise and inverse, with one control point every 4°. We exclusively used 6 MV photons. Collimator rotations of + 10° and −10° were used to increase the modulation possibilities.

**Figure 2:**
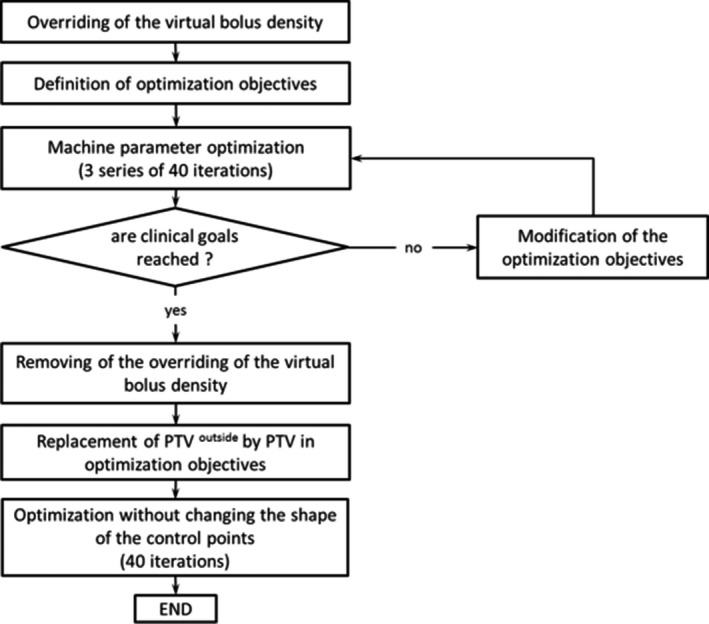
Flow diagram showing the volumetric modulated arc therapy planning process.

**Figure 3:**
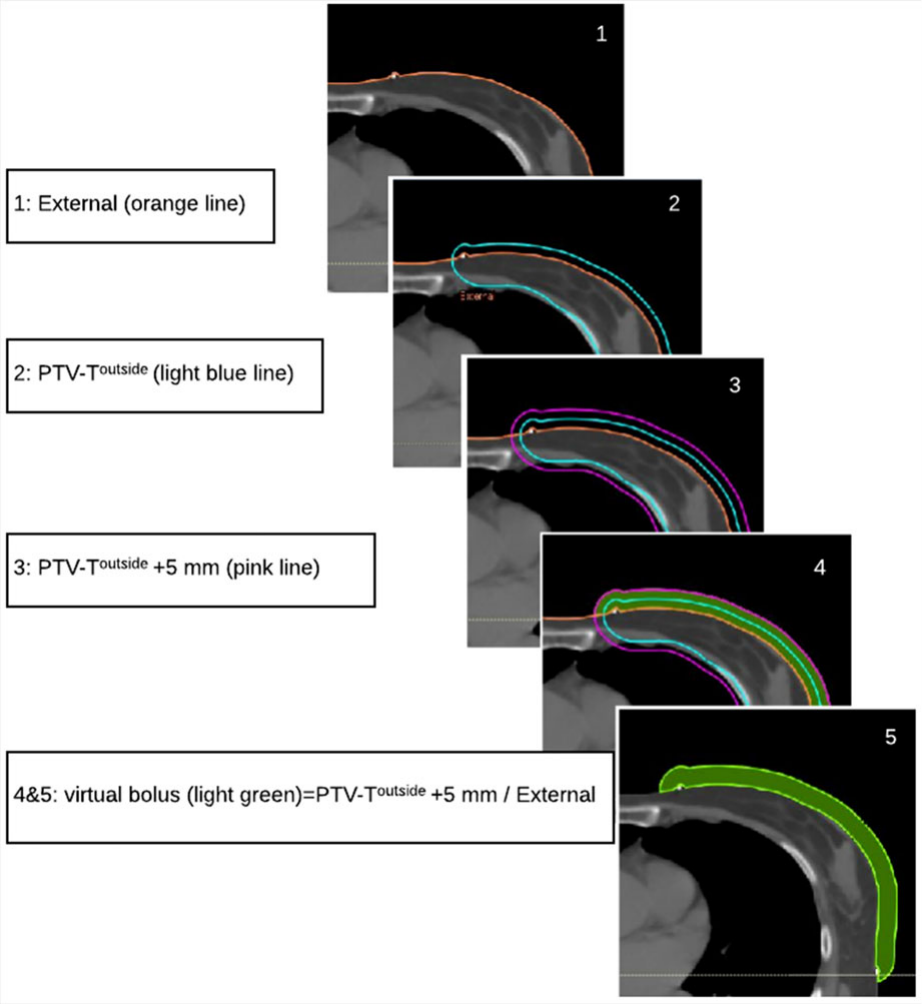
Virtual bolus construction: virtual bolus = (PTV‐T outside + 5 mm) \ external. Virtual bolus thickness = 10 mm.

Inverse planning was made in two steps. The first step of the optimization process was made with the virtual bolus in place (voxel's density of the virtual bolus was set to the density of water). In the second step, the virtual bolus was removed (no density was applied to the virtual bolus) and a new optimization was made without changing the shape of the segments, that is only to adjust the number of monitor units (MU) by control point (Fig.** 2). The bolus construction, shown in Fig. 3, required two steps: first, 5 mm was added at the PTV^outside^. Then, a subtraction was made from the external boundary of the patient automatically generated by the TPS.

During the inverse planning optimization process, objectives were chosen in order to respect the prescription regarding the PTV‐T and fulfill the predefined following clinical goals: D_mean_ < 6 Gy for the heart; V20 < 30% and V30 < 20% for the left lung; and D_mean_ < 3 Gy for contralateral organs (lung and breast). In addition, for healthy tissues, the maximum dose should be inferior to 55 Gy. For planning target volumes, 95% of their volume should be covered by 95% of the prescribed dose (V95%). Prescription was made on the median dose of PTV‐T (50 Gy) for both VB‐VMAT (at the end of the second step of the optimization process) and NoVB‐VMAT. However, for VB‐VMAT, the dose was prescribed on the PTV^outside^ for the first step of the optimization process. The initial optimization objectives were fixed among cases but they could then be adjusted to meet clinical goals. Clinical goals were in accordance with the external RT guidelines published in 2007.^14^ The NoVB‐VMAT plans were reoptimized based on VB‐VMAT optimization parameters.

### 2.C Institutional standard technique: tangential image‐guided radiation therapy technique

Our institutional standard technique used two tangential fields (for the breast) and four additional static fields (for the nodes) as described in previous publication.^15^ Tangential and node fields are constructed from the PTV^outside^ with margins. An overlap of ≤7 mm at the skin between the tangential and node fields is accepted. 6 MV photons are used (or a mixture of 18 and 6 MV photons for large volumes). The IMC field is treated using a combination of photons and electrons. Contralateral OAR are excluded from the primary fields.

The dose distribution to the breast is optimized using a field‐in‐field technique consisting in suppressing overdoses regions by successive segments. The overdoses areas are hidden by 6% levels. The segment size was restricted to a 1.5 cm around the prescription point and a minimum of four MU per segment was required. Three or four segments are usually used; the main segment that corresponded to the whole tangential field consists of approximately 80% of the MU.

### 2.D Comparison criteria and statistics

The main goal of this study was to evaluate the benefits when using a virtual bolus during the planning process to maintain the coverage of target volume when breast shape modifications or set up errors occur. Three steps were used for the demonstration:

(a) First, the VB‐VMAT plans were compared to the NoVB‐VMAT plans. For the CTV‐T and CTV‐N, we have compared target coverage (V95%) as well as homogeneous and conformity index (HI and CI). As mentioned above, for evaluation purposes, the CTV‐T was limited at 5 mm inside the external contour to exclude the first millimeters where the uncertainty on dose calculation is more elevated. The HI was calculated according to the following formula: HI = D2% − D98%/D where D2% is the dose to 2% of the volume, D98% is the dose to 98% of the volume, and D is the prescribed dose. Then, the conformity index (CI) for the combined CTVs was compared. The CI was automatically calculated on Raystation^®^ defined as follows: CI = TV_PVI_/V_PVI_. (TV_PVI_ is the target volume covered by the prescription isodose and V_PVI_ is the total prescription isodose volume.) Dosimetric results on the OAR were also detailed.
(b) The same analysis was performed on CT2 for the second step.
(c) The third step was the comparison of the coverage modifications between the two CT for VB‐VMAT and Tan‐IMRT. For that purpose, we used the V95% data for both techniques and their differential (∆V95% (CT1‐CT2)).
(d) The Wilcoxon signed‐rank test was used for statistical analysis. The significance of the *P*‐value threshold was set at 5%. All plans were performed by the same experienced physicist and improved to meet the clinical objectives.

## 3 RESULTS

**Figure 4:**
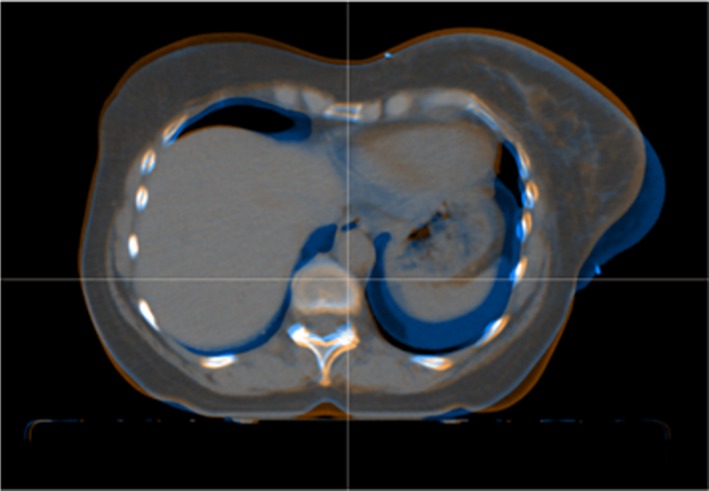
Image registration and breast shape modification during treatment. This figure illustrates conformation modification of breast during radiation therapy. The blue image is a transverse section of CT1 registered with CT2 colored with orange.

Breast volumes on CT1 and CT2 are summed up in Table** 1. The increase in breast volume was not the only cause of modification of the external breast shape. The subject who had the most different conformation between the two CT had a smaller breast volume on the CT2 but the breast's shape was modified (Fig.** 4 shows a breast volume less falling into the inferior and external directions). Breasts during RT develop edema^16^, ^17^ and tend to move into the anterior direction.

**Table 1:** Breast volume and its variation between the two scans

Patients	Breast/chest wall volume on CT1 (cc)	Breast/chest wall volume on CT2 (cc)	ΔVariation of breast/chest wall volume between CT1 and CT2 (%)
1	403	425	+5.0
2	256	288	+11.0
3	555	589	+5.8
4	1450	1427	−2.0
5	639	664	+3.7
6	718	789	+8.9
7	1456	1400	−4.0
8	729	528	−11.5
9	337	427	+21.0
10	426	554	+23.0

Details for each patient on the first scan and a second one during treatment. +ΔVariation, volume increase; − ΔVariation, volume decrease; CT1, first scan; CT2, second scan.

### 3.A Results for the VB‐VMAT and NoVB‐VMAT plans on CT1

The first analysis compared the dose distributions on CT1 between plans optimized with and without the virtual bolus method. No degradation of coverage of target volumes were observed (Table** 2). A significant better coverage for CTV‐T^evaluation^ was found for the VB‐VMAT plans compared to the NoVB‐VMAT plans: V95%‐CTV‐T^evaluation^ was 99.4% [98.5–99.8] for VB‐VMAT compared to 98.4% [96.9–99.6] for NoVB‐VMAT (*P* = 0.037). A better CI was also found for the VB‐VMAT plans with a value of 0.99 [0.99–1] vs. 0.98 [0.97–1] for NoVB‐VMAT (*P* = 0.025). With regard to the OAR, the VB‐VMAT plans did not alter the dosimetric results (Table S1). No difference was found for the mean dose (D_mean_) of the heart nor V30 to the ipsilateral lung (4.6 Gy [2.9–5.8] for VB‐VMAT vs. 5.0 Gy [3–7.6] for NoVB‐VMAT (*P* = 0.375) and 17.1% [13.7–19.1] with VB‐VMAT vs. 17.0% [13.8–18.9] with NoVB‐VMAT (*P* = 0.358), respectively). The D_mean_ to the contralateral lung was found lower for the VB‐VMAT plans (2.5 Gy [2.1–3]) compared to NoVB‐VMAT (2.7 Gy [2.3–3.1]) (*P* = 0.024). The same observation was found for the D_mean_ to the contralateral breast with 2 Gy [1.7–2.3] for the VB‐VMAT plans compared to 2.1 Gy [1.7–2.4] for NoVB‐VMAT (*P* = 0.002). The reported D_mean_ to the skin was higher for VB‐VMAT compared to NoVB‐VMAT (45.4 Gy [44.7–46.1] vs. 44.1 Gy [44.4–44.8] (*P = *0.006)).

**Table 2:** Target volumes coverage on CT1 for VB‐VMAT and NoVB‐VMAT

CT1	VB‐VMAT				NoVB‐VMAT				Wilcoxon's signed‐rank test
	Median	Mean	Min	Max	Median	Mean	Min	Max	
CTV‐T^evaluation^									
V95% (%)	99.4	99.4	98.5	99.8	98.6	98.4	96.9	99.6	0.037^a^
HI	0.07	0.08	0.06	0.10	0.08	0.08	0.05	0.11	0.234
CI	0.99	0.99	0.99	1.00	0.99	0.98	0.97	1.00	0.025^a^
CTV‐N									
V95% (%)	100	99.9	99.4	100.0	100	100.0	99.6	100.0	1
HI	0.06	0.06	0.04	0.07	0.05	0.05	0.03	0.06	0.058
CI	1	1.00	0.99	1.00	1	1.00	1.00	1.00	1

a *P* < 0.05, according to the Wilcoxon's signed‐rank test. CT1, first scanner; VB‐VMAT, volumetric modulated arc therapy using the virtual bolus method; NoVB‐VMAT, volumetric modulated arc therapy without using the virtual bolus method; Min, minimum; Max, maximum; CTV, clinical target volume; V95% (%), percentage volume receiving ≥95% of the prescribed dose; HI, homogeneity index; CI, conformity index.

### 3.B Results for the VB‐VMAT and NoVB‐VMAT plans on CT2

The second step evaluated the benefit of the use of the virtual bolus by comparing the dose distributions computed on CT2 for VB‐VMAT and NoVB‐VMAT.

Table** 3 shows a better coverage of target volumes for the VB‐VMAT plans compared to the NoVB‐VMAT plans. V95%‐CTV‐T^evaluation^ was 98.9% [96.1–99.6] on CT2 for the VB‐VMAT plans compared to 92.6% [85.2–97.7] for NoVB‐VMAT (*P *= 0.0002). The HI was 0.1 [00.7–0.13] for the VB‐VMAT plans compared to 0.13 [0.09–0.18] for NoVB‐VMAT (*P *= 0.006). No significant difference in favor of the NoVB‐VMAT plans was reported on the OAR (Table S2). No difference was found for the mean dose (D_mean_) of the heart nor V30 to the ipsilateral lung (5.3 Gy [3–7.6] for VB‐VMAT vs. 5.3 Gy [3.1–7.7] for NoVB‐VMAT (*P *= 0.769) and 16.2% [13.4–19.0] with VB‐VMAT vs. 16.0% [13.2–18.8] with NoVB‐VMAT (*P *= 0.131), respectively). The percentage of the humeral head receiving 20 Gy and the D_mean_ to the right breast were higher for the NoVB‐VMAT plans (which was already significantly higher for the NoVB‐VMAT plans on CT1). The reported D_mean_ to the skin was higher for VB‐VMAT compared to NoVB‐VMAT (45 Gy [43.9–45.5] vs. 42 Gy [40.5–43.5] (*P* = 0.002)).

**Table 3:** Target volumes coverage on CT2 for VB‐VMAT and NoVB‐VMAT

CT2	VB‐VMAT				NoVB‐VMAT				Wilcoxon's signed‐rank test
	Median	Mean	Min	Max	Median	Mean	Min	Max	
CTV‐T^evaluation^									
V95% (%)	98.5	98.2	96.1	99.6	93.7	92.9	85.2	97.7	0.002^a^
HI	0.09	0.1	0.07	0.13	0.12	0.13	0.09	0.18	0.006^a^
CI	0.98	0.9	0.64	0.99	0.94	0.87	0.85	0.98	0.083
CTV‐N									
V95% (%)	99.5	97.2	87.1	100.0	99.4	97.3	87.4	100.0	0.528
HI	0.07	0.1	0.05	0.29	0.07	0.09	0.05	0.26	0.746
CI	0.99	1	0.87	1	0.99	0.89	0.05	1	0.345

a *P* < 0.05, according to the Wilcoxon's signed‐rank test. CT1, first scanner; VB‐VMAT, Volumetric Modulated Arc Therapy using the virtual bolus method; NoVB‐VMAT, Volumetric Modulated Arc Therapy without using the virtual bolus method; Min, minimum; Max, maximum; CTV, Clinical Target Volume; V95% (%), percentage volume receiving ≥ 95% of the prescribed dose; HI, homogeneity index; CI: conformity index.

### 3.C ∆V95% (CT1‐CT2) for VB‐VMAT vs. Tan‐IMRT

The last step was the comparison of the dosimetric impact of breast modification on CT2 between VB‐VMAT and the institutional standard technique Tan‐IMRT.

The mean percentage volume of the V95%‐CTV‐T^evaluation^ for the 10 analyzed patients of the study for VB‐VMAT were 99.4% and 98.2% on CT1 and CT2, respectively. For Tan‐IMRT, it was 94.1% and 93.1%, respectively. The differential for median values was 0.9% for VB‐VMAT and 0.7% for Tan‐IMRT. There was no significant difference between the median differential of target volume coverage between the two techniques (*P* = 1).

## 4 DISCUSSION

This study is a three‐step demonstration of the benefit and safety of using a virtual bolus during treatment planning for arc therapy for the breast. In this work, a large variety of chest anatomies are displayed, illustrating a range of clinical situations. This is the first study to evaluate the benefit of using a virtual bolus on real target volume modification.

This work first showed that the use of a bolus on the initial planning CT did not alter the dosimetric results neither for target volume coverage neither to the OAR. No significant result in favor of the NoVB‐VMAT plans was noted. Even slightly better results were found for VB‐VMAT. (D_mean_ to the contralateral lung and the breast on VB‐VMAT vs. NoVB‐VMAT were 2.5 Gy [2.1–3] vs. 2.7 Gy [2.3–3.1] (*P *= 0.024) and 2 Gy [1.7–2.3] vs. 2.1 Gy [1.7–2.4] (*P *= 0.002), respectively.) This was also verified on CT2 for the contralateral breast (2.1 Gy [1.8–2.6] for NoVB‐VMAT vs. 2.0 Gy [1.6–2.4] for VB‐VMAT (*P* = 0.002)). The differences in coverage of the external part of the PTV‐T lead the software to choose different ways of optimization between VB‐VMAT and NoVB‐VMAT. This explains the small, but significant, differences between the results even if identical optimization objectives were used.

Second, this paper demonstrated that using a virtual bolus during optimization improves dosimetric results on a second CT required during treatment compared to the NoVB‐VMAT plans. V95%‐CTV‐T^evaluation^ was 98.9% [96.1–99.6] on CT2 for VB‐VMAT compared to 92.6% [85.2–97.7] for NoVB‐VMAT. This difference was significant (*P *= 0.0002).

Finally, this study showed that the degradation of the target volume coverage between CT2 and CT1 for the VB‐VMAT plans was similar to a Tan‐IMRT technique as the median differential of V95%_CTV‐T^evaluation^ between the two CT was 0.9% for VMAT and 0.7% for Tan‐IMRT (*P = 1*). Even if Tan‐IMRT technique gave satisfying dose distribution on CT2, it was still inferior to the VB‐VMAT plans. When looking at the mean V95%‐CTV‐T^evaluation^, VB‐VMAT achieved a coverage of 99.4% on CT1 and 98.4% on CT2, whereas the mean results for the Tan‐IMRT technique were lower (mean V95%‐CTV‐T^evaluation^ = 94.1% on CT1 and 93.1% on CT2). Only the VB‐VMAT plans achieved delivery of 95% of the prescribed dose to 95% of the CTVs on CT2.

We chose to study one of the worst‐case scenarios with respect to complexity of treatment volume, namely the left breast with whole regional nodal irradiation. In this setting, the standard method (Tan‐IMRT) has some limitations, particularly in the region of the junctions of fields, even if a single monoisocenter is used. This explains the dosimetric results below 95% for the CTV‐T^evaluation^ coverage with Tan‐IMRT. These cases with unsatisfying dosimetric results with conventional techniques are good candidates for VMAT.

As expected for the VMAT plans, homogeneity and conformity indexes were very satisfying as mean values approached 0 for HI and even reach 1 for CI.

**Figure 5:**
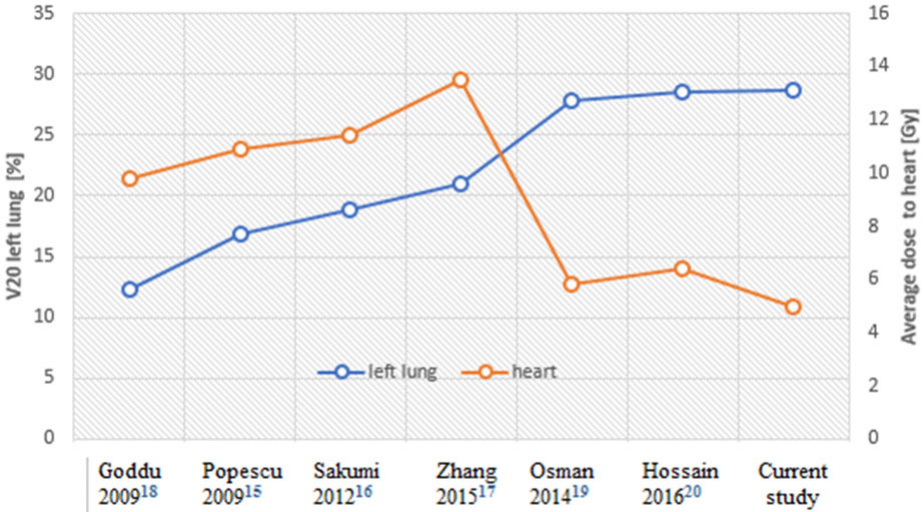
Dependency of ipsilateral lung and heart doses. This figure illustrates a potential dependency of heart dose and ipsilateral lung dose for regional left‐sided breast VMAT RT. The four first studies (on the left) demonstrate V20 Gy < 25% for ipsilateral lung and an average dose (D_mean_) to the heart >9 Gy. In the three other studies (on the right), the protection of the heart is improved (D_mean_ < 6.5 Gy), but is accompanied by an increased the dose to ipsilateral lung.

The comparison of the dosimetric results of VB‐VMAT with published treatment planning studies evaluating free‐breathing arc therapy for the locoregional left‐sided breast (including internal mammary chain)^18^, ^19^, ^20^, ^21^, ^22^, ^23^ are summarized in Table** 4. In all those studies, the same PTV coverage criteria were used: at least 95% of the PTV should receive 95% of the prescribed dose (V95%‐PTV). The published values of V95%‐PTV ranged from 95.2 to 99.4%. Our results lay in the center of this interval. The best coverage results were obtained with Tomotherapy^®^ 21. This may be explained by the overestimation of the calculated dose in the first mm close to the skin by the Tomotherapy^®^ TPS. Zhang et al. also reported excellent results for V95%‐PTV with VMAT but this study included exclusively chest wall and used real bolus.^20^ PTV are typically used in literature to compare coverage on initial plans in order to ensure the CTV coverage for each fraction. In this study, we used CTV‐T^evaluation^ as the CT2 simulates a daily fraction setup for which we intend to evaluate the CTV coverage. Large variations of dose to the ipsilateral lung and heart are observed between the different published results. Fig. 5 illustrates a potential dependency of the V20 Gy to ipsilateral lung and heart doses for a given level of PTV coverage and protection of contralateral OAR. Furthermore, the differences between the results in literature can be mainly explained by the differences in PTV margins. As other authors, we have chosen to reduce the cardiac dose. However, our dose on the ipsilateral lung remained underneath critical dose levels for toxicity. For contralateral organs, doses are more homogeneous between the different studies. As seen Table** 4, we chose to keep doses to contralateral OAR at a very low level. This is indeed a major concern in our plan optimization, particularly for young patients, as it has been shown that second cancer risk is dose dependent and inversely related to patient's age at his first treatment.^24^, ^25^ We found that the D_mean_ to the skin was higher in VB‐VMAT compared to NoVB‐VMAT in both CT1 and CT2 evaluations. Our results are higher than previously published data,^12^ but comparison should take into account the lateral and craniocaudal limits of the skin volume. Moreover, differences may be explained by the uncertainties of calculation in the first mm. Indeed, TPS with collapsed cone algorithms, do not provide accurate dosimetry in the first millimeters.^26^, ^27^, ^28^ As stated in the AAPM TG 176 report, the depth of the sensitive basal layer ranges from 0.05 to 0.4 mm deep.^29^, ^30^ ICRU and ICRP selected 0.07 mm as the reference depth for the skin.^31^ Thus, measurements made at an effective depth greater than the basal layer depth (such as 5 mm in this study or recent publication^12^) will overestimate the “skin dose.” The accuracy of superficial dosimetry depends on the dose calculation grid size. In this study, the grid size was 3 mm which is much bigger than the reference depth for the skin.

**Table 4:** Dosimetric results from different planning studies

	Goddu Tomotherapy^®^ 2009^21^	Popescu VMAT 2009^18^	Sakumi VMAT 2012^19^	Zhang VMAT 2015^20^	Osman VMAT 2014^22^	Hossain VMAT 2016^23^	VB‐VMAT
PTV							
V95% (%)	98.0	98.2	97.8	98.5	95.4	NA	97.3
Ipsilateral lung							
D_mean_ (Gy)	9.6	11.6	12.7	12.8	14.0	17.7	15.1
V5 Gy (%)	57.2	70.2	74.6	61.1	67.5	NA	70.7
V20 Gy (%)	12.3	16.9	18.9	21.0	27.9	28.6	28.7
Contralateral lung							
D_mean_ (Gy)	2.1	2.9	4.0	4.49	3.4	6.0	2.5
Heart							
D_mean_ (Gy)	9.8	10.9	11.4	13.5	5.8	6.4	4.6
Contralateral breast							
D_mean_ (Gy)	3.1	3.2	3.1	1.7	2.8	3.6	2.0

PTV, Planning Target Volume; Vn (%), percentage volume receiving ≥nGy; D_mean_, mean dose; VMAT, volumetric modulated arc therapy.

The clinical impact of such technique will still need to be evaluated. Studies reporting the clinical results of VMAT‐based breast treatment demonstrated low toxicity with optimal local control.^6^, ^7^, ^8^, ^9^, ^10^ The study reporting the outcome of stage III breast cancer treated with VMAT including the IMC in locoregional nodes reported higher doses to ipsi‐ and contralateral OAR than ours; however, their 2‐year toxicity was low with no severe cardiac nor lung toxicities.^11^ Prospective studies with toxicity analysis and long‐term follow‐up are needed.

The main limitation of this study is the relative small number of cases. However, to our knowledge our study is the first to report dosimetric results of VMAT plans with an evaluation of interfraction motion based on real observations of modified anatomies during the course of treatment. Another intricacy of this study is the method used for isocenter reposition to compute the dose distributions on CT2. The rigid registration method used translations but no rotation in order to reproduce online fusion which therapists would be performing on cone beam CT (CBCT) during the radiation course.

VMAT improves dose homogeneity and conformity for locoregional breast radiotherapy, but, due to the small and complex shape of the segments, their dose distribution may suffer more from intra‐ and interfraction motion. In this study, using a virtual bolus, we focused on taking into account interfraction movements. Indeed, a systematic review covering 3378 studies concluded that interfraction motion is larger than intrafraction^32^ which was confirmed in another recent study.^33^

Interfraction motions are due to setup errors (random component) and conformation modifications (systematic component). Concerning setup errors, improving contention has always been a concern in radiation oncology: two studies^34^, ^35^ concluded that the use of a “*Posi‐Rest*” minimized the setup errors by 10 mm, whereas another team only report the more comfortable aspect of a personalized contention.^36^ Some teams tried to use thermoformed masks but it seemed to mimic a bolus effect, increasing the skin toxicity. Tested for breast treated with Tomotherapy^®^, thermoplastic mask did not improve the setup reproducibility. In the present study, the registration between CT1 and CT2 revealed well known setup errors as the position of the arms. Surface‐based imaging systems may help with this common issue for breast RT. The position of the head (chin tilt and twist) was also hardly perfectly reproducible. However, as inversed planning for VMAT allows to use specific constraints on central OAR, the need to twist the chin is no longer required. Patients may be treated with their chin straight, which is more easily reproducible. Image‐guided radiotherapy (IGRT) with daily checked CBCT would get rid of some of the interfraction motion by detecting setup errors. A study comparing daily CBCT and portal images (PI) showed that PI underestimated positioning errors of 20–50%.^24^ That observation speaks up for the use of CBCT in IGRT for breast RT. Concerning conformation modifications, the virtual bolus technique, first described by Giorgia et al,^12^ is now used in recent published series about arc therapy for breast to take into account setup errors and glandular modifications: edema, volume diminution, conformation modification.^8^, ^20^ Other techniques based on additional offset have recently emerged.^37^ Finally, adaptive RT provides of course another way to deal with this problem.

Intrafraction motion is mainly due to breathing movements but also to relaxation of the patient during the session.^38^ It was confirmed by our own experience with surface‐based repositioning system. The relaxation motion can be corrected by monitoring the position of the patient during the session with surface‐based imaging systems like Align‐RT^®^. Interplay effect between breathing and MLC motion can degrade the dose distribution. This effect has been studied in only two publications for tangential fields techniques^39^, ^40^ but not for VMAT for breast. However, the interplay effect has been largely studied in the case of lung RT^41^, ^42^, ^43^, ^44^, ^45^, ^46^ with motion amplitude being largely superior to those encountered in breast treatment. Those studies show that the interplay effect generates a blurring effect on the dose distribution. Thus, as intrafraction motion of the breast due to breathing is small (of the order of a few mm^25^), low impact on the dose distribution is expected in the setting of breast RT. Anyway, breathing control has been used to minimize heart dose but it may also reduce uncertainties due to breathing movements. Several studies with arc therapy have been published.^22^, ^47^, ^48^, ^49^ The team of the Oncology Institute of Southern Switzerland reported the first preclinical experience for breast treatment with VMAT and breathing control.^50^

## 5 CONCLUSION

The safety and the benefit of a virtual bolus method were confirmed by this study for real patients with breast modifications occurring during the course of radiotherapy treatment. The use of a virtual bolus significantly improves the coverage of CTVs during the treatment fraction compared to technique which does not use it. Similar and even slightly better dose distribution are obtained when using a virtual bolus during the planning process. On a logistic level, although requiring two stages in planning, this technique is less time‐consuming than the previous standard field‐in‐field technique and is used in routine practice in our department for breast treatment including locoregional lymph nodes for patients over 50 years old.

## CONFLICT OF INTEREST

The authors declare no conflict of interest.
