# Supplementary figures and images for: Safety and benefit of using a virtual bolus during treatment planning for breast cancer treated with arc therapy
Source: J Appl Clin Med Phys. 2018 Jun 30;19(5):463–72. doi: 10.1002/acm2.12398 (PMC6123145; doi:10.1002/acm2.12398)

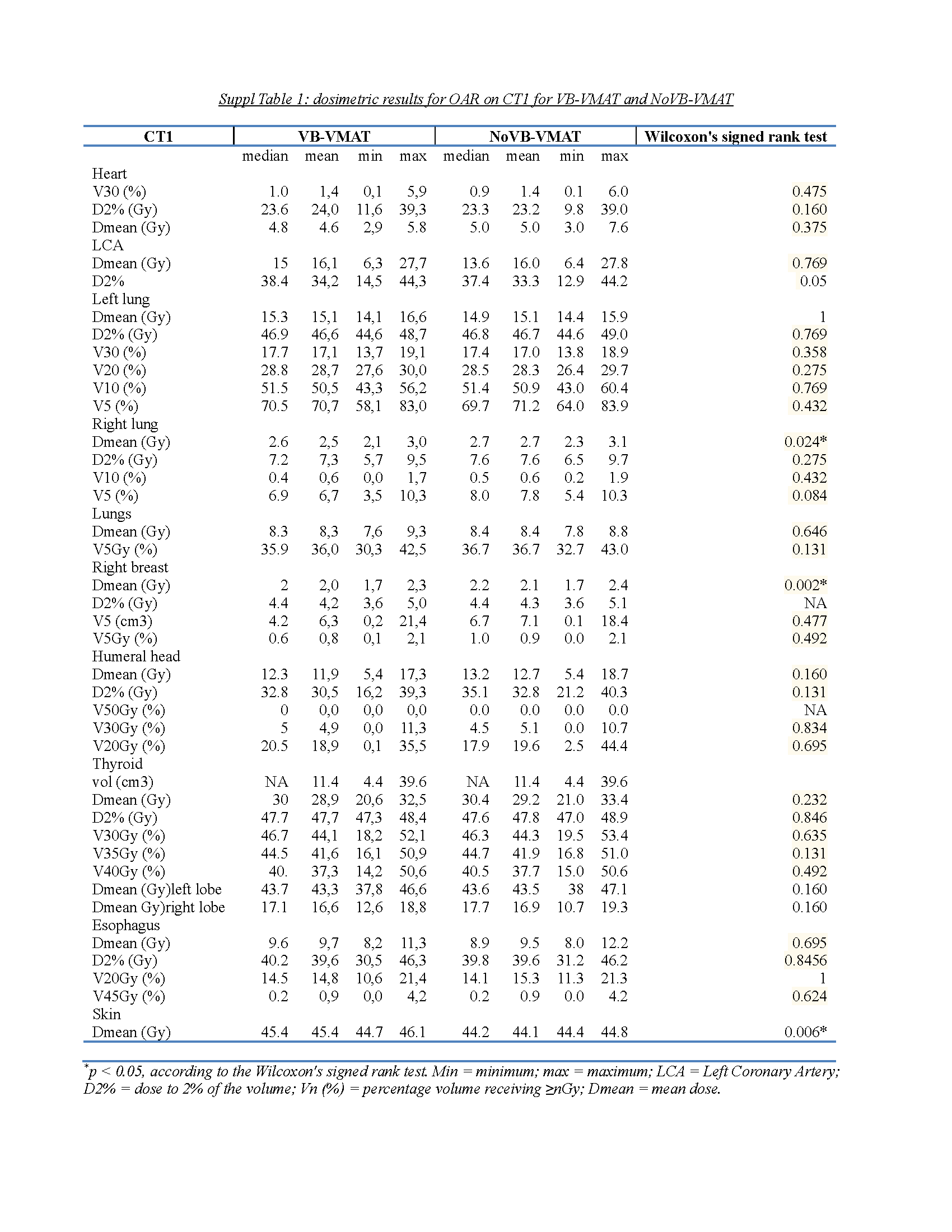

Supplement: Supplementary file 1 [file ACM2-19-463-s001.png]

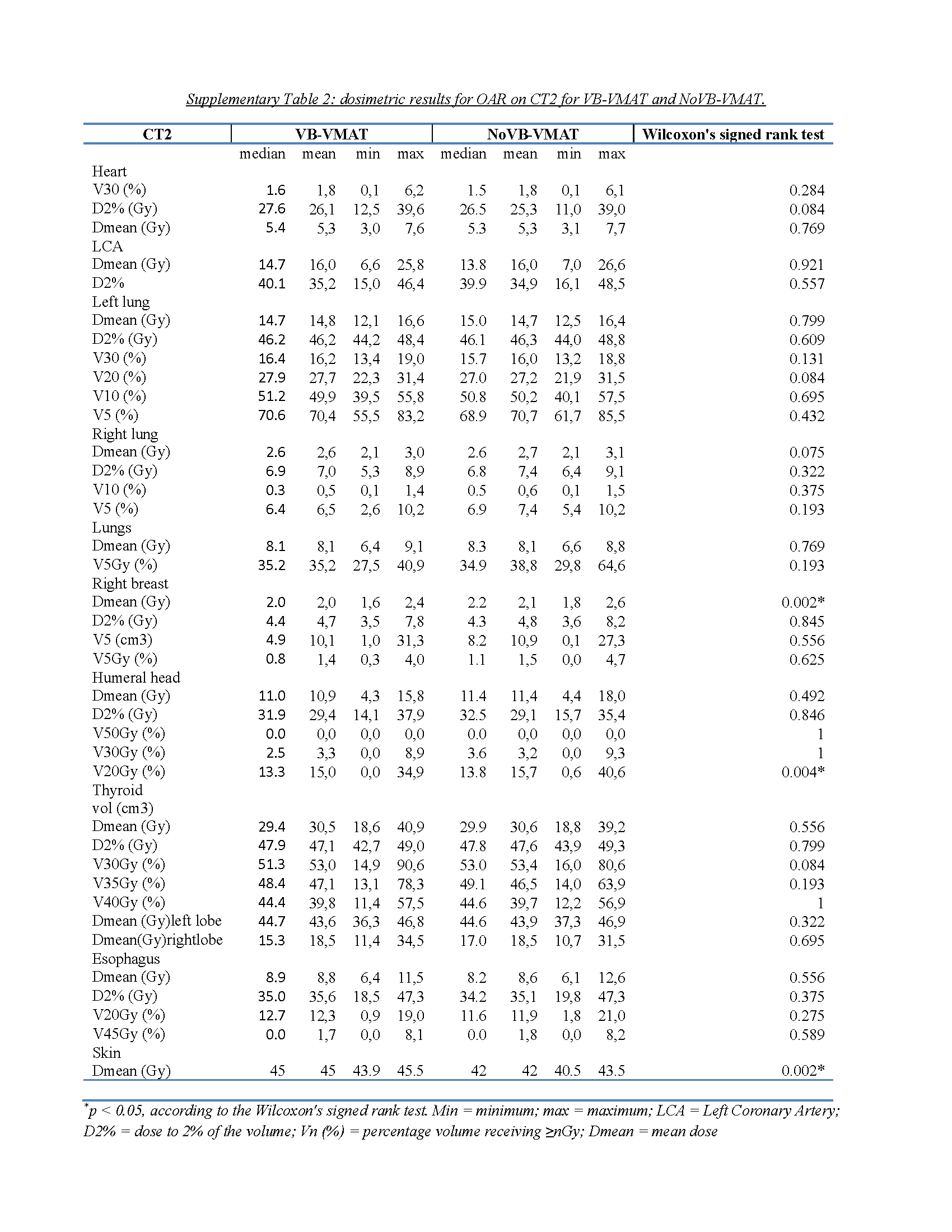

Supplement: Supplementary file 2 [file ACM2-19-463-s002.png]
